# Codeine Addiction and Internet Forum Use and Support: Qualitative Netnographic Study

**DOI:** 10.2196/12354

**Published:** 2019-04-25

**Authors:** Eleanor Lee, Richard J Cooper

**Affiliations:** 1 School of Health and Related Research University of Sheffield Sheffield United Kingdom

**Keywords:** codeine, addiction, abuse, opioid, internet, prescription, over-the-counter

## Abstract

**Background:**

The use of codeine as an analgesic is well-recognized, but there are increasing concerns that for some individuals continued use may lead to misuse, dependence, and fatalities. Research suggests that those affected may represent a hard-to-reach group who do not engage with formal treatment services.

**Objective:**

This study sought to explore the experiences of people with self-reported addiction to codeine and, specifically, how a social media forum is used to communicate with others about this issue.

**Methods:**

Using a qualitative netnographic methodology, the social media forum Mumsnet was used, with permission, and searches were undertaken in 2016 of any posts that related to codeine and addiction. A total of 95 relevant posts were identified; a purposive sample of 25 posts was selected to undertake subsequent six-stage thematic analysis and development of emerging themes. These 25 posts were posted between 2003 and 2016 and comprised 757 individual posts.

**Results:**

Individuals created posts to actively request help in relation to usually their own, but occasionally their partner’s or relative’s, problems relating to codeine use and self-reported “addiction.” Varying levels of detail were provided in narratives of problematic codeine use. There were both positive and negative descriptions of side effects emerging, problems experiencing withdrawal, and failed attempts to discontinue codeine use. Mainly positive and supportive responses to posts were identified from those with either self-reported health profession experience or lay respondents, who often drew on their own experiences of similar problems. Treatment advice emerged in two main ways, either as signposting to formal health services or to informal approaches and often anecdotal advice about how to *taper* or use *cold turkey* techniques. Some posts were more critical of the original poster, and arguments and challenges to advice were not uncommon. Shame and stigma were often associated with users’ posts and, while there was a desire to receive support and treatment advice in this forum, users often wanted to keep their codeine use hidden in other aspects of their lives. Distinctly different views emerged as to whether responsibility lay with prescribers or patients. Some users expressed anger toward doctors and their prescribing practices.

**Conclusions:**

This study provides a unique insight into how a public internet forum is used by individuals to confirm and seek support about problematic codeine use and of the ways others respond. The pseudonymous use of internet forums for such information and variation in treatment options suggested by often lay respondents suggest that increased formal support and awareness about codeine addiction are needed. There may be opportunities for providing further support directly on such online forums. Improvements in prescribing codeine and in the over-the-counter supply of codeine are required to prevent problematic use from occurring.

## Introduction

Opioid medicines are an important group of medicines that are widely used for the treatment of pain [1]. Their use is also increasing; in the United Kingdom, there has been a five-fold increase in their use between 1991 and 2009 [2]. This trend has increased concerns about possible opioid harms, such as dependency and addiction. In England, there has also been an increase in the number of deaths caused by codeine, rising from 131 in 2016 to 156 in 2017 [3]. Globally, estimates of opioid addiction have varied or are lacking in many countries, but data in the United States suggests that between 8% and 12% of patients may be addicted [4]. Patients affected may not present, or be referred, to specialist addiction clinics [5,6], and the number of individuals treated may be an underestimate and reflect a “hidden” population [6]. Of those who presented to formal drug services in England in 2016-2017, 1618 patients were treated where a prescribed opioid, and no illicit substance use, was identified; a further 554 sought treatment involving nonprescription or over-the-counter opioids (information was acquired via a freedom-of-information request sent to Public Health England in July 2018 regarding over-the-counter and prescription medicine data from the National Drug Treatment Monitoring System). Research is inconclusive about the profile of those affected, although some studies have identified females as being more likely to misuse over-the-counter medicines [7]. It should also be noted that a number of terms are used to describe problematic use of medicines, reflecting different clinical and social manifestations and interpretations related to this issue [7]. Current clinical terminology relates to substance use disorder of differing severity and subtypes [8]; however, in this paper it will be argued that *addiction* is justified methodologically as a lay term, but *misuse* is justified as a more neutral term when presenting findings.

Codeine is one of the most frequently supplied opioids; it is supplied via prescription but also over-the-counter from pharmacies in some countries [9]. Codeine is a relatively weak analgesic and is commonly coformulated with other analgesics, such as paracetamol and ibuprofen. In addition to the potential for dependence and addiction to codeine, these compound formulations are associated with incidents of hepatotoxicity and gastric bleeding [10,11]. This has led to examples of upregulation of codeine; in Australia, the drug was recently removed from pharmacy sale to prescription-only use [9].

Support and treatment for those affected by problematic use of codeine is available from several sources [6], ranging from formal drug and alcohol treatment service, group support such as Narcotics Anonymous, private clinics, involvement of general practitioners, online groups, and self-treatment. Those affected have been recognized as a hard-to-reach group [6,12], who may not want others to know about their misuse and so may not present to formal treatment services.

The role of the internet in relation to medicine misuse represents an emerging area of concern. Research has explored how individuals communicate and discuss narratives of codeine misuse and experimentation. This has been identified through written posts about codeine *trips* [13] and *glamorizing* codeine misuse through images and specific media, such as Instagram [14] or Twitter for illegal prescription opioid sales [15]. Such insights do not capture how the internet is used to seek support for misuse of medicines such as codeine. Using the internet in this way represents one aspect of the wider topic of how different online resources are used by the public to obtain information and support about their health and illness. A total of 49% of individuals surveyed in 2015 in Great Britain reported searching for health-related information in the previous 3 months, compared to only 18% in 2007. Searching for disease-specific information has also been identified [16], particularly to enable diagnosis [17,18]. A recent survey found the internet to be the most common source used for seeking help for codeine use (12%) compared to only 3% of respondents using their doctor in the United Kingdom and Ireland [19]. Historically, specific online groups such as Codeinefree.me and Over-Count [20] have provided support for prescription and over-the-counter medicines and may be important for confirming self-reported “addiction” and for gaining more information [6]. Such online sites are private and represent only a minority of online opportunities to obtain help and support, including many general social media sites. Social media sites encourage discussions and have been argued to be particularly helpful in the “absorption of knowledge” [18]. An associated concern, however, relates to the quality of information on such sites, although it has been noted that *counterbalancing* *comments* can correct incorrect information [18]. Regarding wider substance misuse, the use of online resources such as social media has been recognized as being important in relation to digital recovery [21,22]. Nearly half of a sample of US individuals receiving substance misuse treatment felt that social media content triggered cravings but around half also reported beneficial recovery information on social media [22].

The primary aim of this study was to explore how a social media discussion forum was used to discuss self-reported codeine addiction. Further aims were to understand the types of information and support provided by others and any self-reported harms related to codeine.

## Methods

A qualitative netnographic methodology was used to explore this topic, involving the selection and analysis of online posts and interactions and, in particular, differences between core and peripheral contributors [23]. Mumsnet [24] was chosen as the host forum site as it represents a popular site that has been used in previous research and presents posts and associated threads publicly [18], avoiding any ethical concerns about *lurking* on private forums. Mumsnet was approached and permission was gained from the website to use their data.

Ethical approval was obtained from The University of Sheffield. Consent was not obtained from those contributing to posts based on current guidance as to the use of online social media posts. However, to maintain anonymity, users’ names will not be presented, and all quotes will be paraphrased having been checked in Mumsnet and Google that they cannot be used to identify the thread. Evidence on a specific link between parenting and misuse of medicines is scant, but groups such as pregnant and parenting women have been considered particularly concerning in relation to opioid misuse [25] and effects on children [26].

Data collection involved accessing the Mumsnet site and using its standard search function. A variety of search terms were trialed based on previous reviews of the literature [7]; the phrase “codeine addiction” was eventually selected, as using other search terms resulted in too many nonsalient threads or too few threads. Using “codeine addiction” resulted in a total of 141 threads being identified during searches in July 2016; however, on subsequent checks, only 100 of these were available to search further, which was thought to have resulted from original posts having been removed. Of these 100 resulting threads, 5 (5.0%) were excluded as they were surveys, advertisements, or links back to other threads. Of the 95 remaining, a purposive sample of 25 threads (26%) were selected based on variables described in Table 1, such as year of posting, whether the original person posting did so about themselves or on behalf of another, involvement of prescription or over-the-counter medicines, and previous Mumsnet research [27]. Excluded threads included predominant reference to noncodeine medication (eg, tramadol addiction); questions about side effects of codeine; and concerns, but not actual experience, of being prescribed codeine.

Posts from each of the 25 threads were extracted and converted to electronic offline text documents. These data were then analyzed initially by one of the researchers (EL), using Braun and Clarke’s six phases of thematic analysis [28]. These phases are linear, although each can be revisited throughout the process. The six phases are as follows: (1) data familiarization, (2) generating initial codes, (3) searching for themes, (4) reviewing themes, (5) defining themes, and (6) writing up. We began this process with immersion in the data in order to become familiar with the dataset, using printed versions of each thread. On first reading, initial thoughts and notes were annotated. Coding by hand provided reassurance of comprehensive coding, even though it would be more difficult to search for and find codes within the data compared to using computer software [28]. When coding the threads, notes were written on paper for ease. A challenge with coding in this study of forum posts was to retain the context, especially when the text is split into fragments [29]. Initial coding was open, also referred to as semantic by Braun and Clarke, which involved attempts to provide literal and descriptive codes related to sections of threads. Following this, latent coding was undertaken, which involved searching for underlying ideas and conceptualizations. After the initial codes were decided, they were grouped into overarching themes to categorize all the data. Next, coding was repeated to ensure that the analysis was complete. The data were reread to confirm that the themes were accurately depicted and reviewed. This ensured that the dataset was well-understood and that there was no missing information. Analysis was initially undertaken by one researcher (EL); another researcher (RJC) then selected a sample of threads and undertook independent coding and theme generation using the same process with printed copies of threads and hand-annotation. Subsequent comparison and discussion of emerging codes and themes were undertaken. A high level of agreement was found based on the sample analyzed and, consistent with the constructionist epistemological basis [30] of qualitative research, evaluation of interrater reliability was not undertaken. Respecting the anonymity of the Mumsnet users, respondent validation was not undertaken.

**Table 1:** Summary of key themes and related subthemes.

Theme	Subtheme
Original post and requests for help	Original reason for codeine use Requests for help Anonymity and identity Acquisition of codeine
Side effect highs and lows	Positive effects of codeine Negative effects of codeine Withdrawal side effects
Support and treatment advice variation	General support Lay and professional advice Specific treatment advice Anecdotal: weaning and cold turkey Formal services
Stigma and shame	Comparison to illicit drug users Hiding misuse from others
Responsibility and blame	Individual and health professional responsibilities
Absent endings	Progress for the original poster How other users have progressed

## Results

### Overview

Analysis of the 25 threads revealed that they originated from various requests for help and guidance based on the narrative summaries provided. These included recurrent themes of side effects, both negative and positive, and strategies used to resolve problematic use and a variety of often negative emotions, such as stigma, shame, and embarrassment from those creating threads. Responses to original posts often offered support based on the lay responder’s similar experiences and associated empathy; responses also offered anecdotal suggestions to either seek formal health care treatment or use informal means of stopping codeine use (see Table 1).

Threads varied considerably in terms of characteristics, with the number of posts varying between 3 and 202; some posts were active for only a few days, but in some cases additional contributions were identified weeks or years later. Thread titles also varied and were often quite dramatic in tone, succinctly summarized the problem, and often posed a question. The majority of sampled posts related to individuals seeking help for themselves, but some involved a partner or relative (see Table 1). Threads also varied in terms of tone, style, interactions between posters, and the content and opinions of the posters. Contributors frequently used specific Mumsnet abbreviations such as OP (ie, original poster), DP (ie, dear partner), or XP (ie, ex-partner) to refer to others; they also used common phrases such as TBH (ie, to be honest) and AIBU (ie, am I being unreasonable), suggesting familiarity with website conventions. A recurrent theme was deleted posts across the threads; it was not possible to analyze such content or understand why posts had been deleted. One instance was identified where a Mumsnet moderator had become involved, but it was not clear why this had happened. The emerging themes are described in more detail in the following sections; participant names are not presented, and illustrations of quotes are paraphrased. Although this study sought insights into those who expressed self-reported “addiction,” the less pejorative term *misuse* will be used in the findings.

### Original Post and Requests for Help

Threads usually involved a combination of descriptions of a problem involving codeine in the original post and subsequent requests for help and support. Original posts contained narratives that varied in the level of detail but often used emotive language and captured aspects such as why codeine had been used; how it was obtained; side effects experienced; and, for some but not all, what had been done to manage problematic codeine use. The latter reflected an important temporal aspect of the original posts, indicating the current situation of the person using codeine; this appeared to vary, with some reporting a problem that had recently been identified and others describing problems over a much longer time period. The excerpt below illustrates how the original post summarized the latter:

I’m struggling to say this, but I’ve got a painkiller addiction involving codeine. I really would like to get help but I’m unsure of how to do this. It’s been going on for over three years now and is getting worse. I have to take two as soon as I wake and use two main brands, and this keeps on happening all day. I have attempted to stop two times before last year, but sickness and bad headaches stopped me. I am aware I am causing myself harm and know I should stop, but how? Ideas of how to stop would be really appreciated.

Thread 12, Original Poster

This was typical of almost all posts in ending with a request for help. It also illustrates how users appeared to have an awareness of a problem, specifically addiction, and how those responding to posts offered similar and often candid reflections of themselves, as in the quote below:

My name is...and I know I’m an addict. It’s not an addiction to alcohol or street drugs and I’ve never smoked, but I am starting to realize I’ve got an addiction to painkillers.

Thread 6, Original Poster

Concerns about anonymity were common; users referred intentionally to *name changing* and altering their usual forum username to another to avoid being recognized. Some original posters used terms such as *ashamed*, *worried*, or *embarrassed* to describe themselves in pseudonyms. Original posters referred to using such online forums for help and support as they did not feel they had support and encouragement in other aspects of their lives:

My husband is very angry about this...I am so ashamed of this, I can’t talk to anyone about it.

Thread 1, Original Poster

The majority of forum users stated that they had originally started taking codeine due to a problem that had required a health professional interaction. They described codeine use being initiated from a doctor’s prescription or from receiving advice from a pharmacist to take codeine combined with ibuprofen or paracetamol. The former presentations reflected conditions such as musculoskeletal problems and obstetric complications; the latter included more self-limiting conditions:

I’d been using pharmacy painkillers that have codeine in them for a long time. I used them occasionally to begin with but then regularly. If I had things like period pain or maybe a hangover, I knew the Nurofen Plus or Solpadeine would make things better.

Thread 8, Original Poster

For some, codeine appeared to have been used alongside several other analgesics, but only occasional references were made by original posters and respondents about other addictions, such as dependency to alcohol or tobacco, and even less frequently about illegal substances. The doses consumed were only mentioned in some posts and respondents did not often seek such information. A recurrent theme in posts related to how codeine was acquired; for some, this was merely descriptive and involved reference to different durations of prescribing. Other accounts involved alternative routes to obtaining codeine, such as the use of multiple pharmacies or ordering online to avoid suspicion or detection:

My partner has been getting lots and lots of codeine in over-the-counter Nurofen Plus over the years by going to different pharmacies.

Thread 7, Original Poster

I’ve also bought it online via online pharmacies. It’s the branded stuff I buy online and from trusted NHS [National Health Service]-approved online pharmacies, so it’s not fake.

Thread 23, Original Poster

### Side Effects of Codeine: Highs and Lows

A second key emerging theme related to the duality of side effects related to codeine use. Positive effects were reported not only in terms of codeine’s ability to relieve pain and allow individuals to function, but also in relation to perceived positive side effects, such as improving mood, inducing euphoria, and reducing anxiety. These were also associated with a perceived increased ability to cope. Posts invariably included more negative aspects related to negative side effects. These varied in type and intensity but included constipation, headache, sickness, lethargy, stomach pains, and a lack of concentration. The following original post illustrates this in relation to contrasting codeine side effects and the impact on the poster, particularly at work:

I really liked the haziness, and the pills helped with my stress and pain...but at work it meant I made little mistakes and couldn’t focus. It wasn’t that bad, but people thought I was lazy.

Thread 8, Original Poster

Requests for help often arose because of difficulties managing negative side effects of codeine. These were particularly acute in relation to withdrawal symptoms when individuals had recognized the need to reduce or stop their use of codeine. These were described by users who had attempted to stop codeine previously as well as by the original poster, as they often updated their thread with their progress. Side effects reported in relation to codeine withdrawal included headaches, gastrointestinal problems, flu-like symptoms, and general malaise:

I’ve now started to feel lethargic in the mornings and been sick quite a lot. In the last couple of weeks, I’ve tried to stop taking them, but by the end of the day I get stomach cramps and I sweat. I feel sick and have a massive headache.

Thread 4, Original Poster

### Support and Treatment Advice Variation

In every thread, at least one user offered advice and support to the original poster but, as noted, the total number of respondents and posts varied considerably. Analysis did not reveal any obvious pattern as to whether aspects of the original post led to more or fewer responses. On most occasions, multiple users wrote positive comments even if they had no knowledge on the topic, as typified by a respondent:

Nothing that new to add, just wanted to give support.

Thread 2, Respondent

Support responses could be distinguished from specific treatment advice, provided more general encouragement, and relied on psychological or emotional responses to try and reassure the original poster:

Don’t feel ashamed. You can deal with this with help. It’s good that you want to do something about it though.

Thread 9, Respondent

In contrast, other users gave more opinionated responses and warnings, which appeared to be intended to shock the original poster and to encourage action:

It is codeine you are addicted to and the excessive paracetamol and also ibuprofen that will have potentially disastrous effects.

Thread 8, Respondent

The other category of response related to more specific treatment advice. Two distinct categories of treatment advice were apparent, involving either self-treatment or engaging with treatment services. Of the former, two main types emerged relating to either *tapering*, involving reducing the dose of codeine gradually over a period of time, and using the *cold turkey* method *,* which involved an abrupt cessation of codeine. Some users gave experiences of both options and how to carry out such methods, such as involving supportive people and indicating how to gradually reduce the dose over time. Such advice was often experiential and appeared to be based on a respondent’s own attempts or from those they knew:

I would try to stop by going cold turkey, but with the withdrawal it was too much. I had sweats, chills, and fatigue...

Thread 23, Respondent

You can wean yourself off them. I was taking a similar amount to what you were taking or more, and I managed to stop after a couple of weeks. You might find things are better without them, it does make you feel quite gloomy.

Thread 11, Respondent

Other users suggested alternative medicines to assist with stopping codeine; examples included using essential oils, acupuncture, exercise, and yoga. Again, these appeared to be suggested based on experiential success by those offering the advice. However, some advice appeared to be more controversial and triggered more opinionated exchanges and responses from others:

Smoking cannabis helps a lot with any addictions, and me and lots of others think it’s a godsend.

Thread 3, Respondent 1

Be careful with rubbish advice. There are others asking for help who are vulnerable.

Thread 3, Respondent 3

Some respondents self-identified as having a health professional background and offered varying levels of detail and advice. At times, this professional knowledge and insight was used to influence or guide a thread in terms of correcting previous advice given, as typified in this exchange:

No doctor who is worth their salt will prescribe methadone for codeine detoxification and especially not if they mention feeling suicidal.

Thread 7, Respondent 1

Many users, including professionals, replied to this message:

Doctors actually do prescribe methadone for codeine dependency.

Thread 7, Respondent 3

I’m a doctor in drug work, and I’d treat this person with methadone or Subutex. Overdose risks are managed by having a supervised consumption regime...

Thread 7, Respondent 4

More commonly, though, original posters were advised to seek additional professional help beyond the forum. In some cases, this related to advice about where to seek help from other online forums where benefits were suggested, often allowing anonymity to be retained. In other responses, advice was given about other services such as Narcotics Anonymous and specialist drug services:

I would suggest getting help with Narcotics [Anonymous]. I think they will treat your difficulties more seriously than your GP [general practitioner].

Thread 9, Respondent

### Stigma and Shame

Two related and negative aspects of codeine misuse emerged in relation to concerns about it being stigmatizing as well as resulting in shame. Stigma often arose when comparisons were made between codeine and illicit drugs users. There was a perception that codeine misuse shared similarities to the use of illicit drugs such as heroin, despite being legal, and carried some of the stigmatizing negative societal views of illicit drug addiction:

There’s a huge problem with heroin where I live, and the thought of queuing next to heroin users in my pharmacy fills me with horror. I suppose I’m not so different to them and it’s just that my little addiction is a legal one.

Thread 8, Original Poster

References to being ashamed were very common in posts and, as noted previously, this arose in relation to changed usernames that contained such wording. In addition, a common manifestation of shame related to a desire to keep addiction secret from family members, doctors, and friends. Users expressed concern about the consequences of their addictions being on medical records, especially when they had responsibilities such as children and jobs. Others did not want to seek help from their doctor due to perceived negative consequences:

If I go to my GP and admit it, what will happen? Will they take my baby away?

Thread 4, Original Poster

I’m scared of going to my GP because I work with his wife and it will go down on my medical record.

Thread 6, Original Poster

Codeine addiction appeared to place a strain on relationships with partners and family members. On multiple occasions, users became upset and expressed worry about the impact of addiction on their children, as well as the risk that their children may be taken away from them.

### Responsibility and Blame

A further negative cluster of themes concerned views about who was perceived to be responsible for codeine addiction and where blame lay. Views differed and some arguments arose between individuals about this issue. Some commented that the person taking the codeine should be responsible for their own actions:

If I’d known about the addiction risk, I would have kept away from them [the drugs]. I don’t think the surgery was to blame, by the way. Not at all...I think it was my naivety blaming the surgery, by the way, not at all, it was me being naive.

Thread 24, Original Poster

I feel sorry for you...[quotes question from original poster about whether anyone has enough time to read leaflets]. Anyone with any sense? You’re arguing that no one warned you, but the warnings were there each time you took medicine from the packet.

Thread 9, Respondent

You were prescribed drugs and foolishly did not take personal responsibility, just as any addict does not.

Thread 9, Respondent

In contrast, others identified blame as arising with doctors for not giving proper advice and information about medicines with the potential for abuse and addiction:

I wish that I’d not taken them at the start...it does make me question whether medical professionals should monitor this more...before it gets out of hand.

Thread 11, Original Poster

### Absent Endings

The final theme concerned the relative absence of updates within threads about how the original poster’s situation had developed over time, even in those with many posts and over long time periods. Few users posted progress updates, but these were often only in the short term. A characteristic of all posts was the distinct absence of resolution or closure about the situation described in the original post. The following quote illustrates a post when an update was provided:

I didn’t take any today, but I still feel terrible. I find it hard to sit because my legs really hurt and I’m very nauseous and have pain everywhere.

Thread 6, Original Poster

Where such progress was reported, other users offered their continued support, and sometimes other users posted at a later date asking about the original poster’s situation. Some respondents did describe their own experiences; in these, there was often more of a sense of finitude in contrast to the original post. These tended to be historic and were often also positive experiences that respondents offered as encouragement, linking to the earlier themes around support.

## Discussion

### Principal Findings

This study has provided a unique insight into how an internet forum is used by individuals to seek and receive help and support in relation to self-reported addiction to codeine and of the harms that can occur. This sample of Mumsnet users appeared to be candid, in terms of both the degree to which original posters offered often highly personal but anonymized accounts of problematic codeine use, as well as from those responding and offering their own experience and opinions and even challenging others. Self-reported addiction to codeine appeared to involve considerable anxiety for individuals, and side effects were a particular concern.

### Limitations

Study limitations relate to the use of only one medicine, codeine, and one internet forum; it is possible that other medicines and forums may have different user profiles and narratives [18]. The term *addiction* used in relation to codeine was self-reported; doses consumed were often not stated so may not reflect a clinically recognized addiction. User profiles were anonymous, so details relating to gender, age, and socioeconomic status could not be ascertained. As noted by Rier [31], the veracity of accounts provided could not be checked, nor did this study specifically seek to analyze the quality of information provided [18]. Further research is suggested involving other medicines and other sites.

### Comparison With Previous Work

Several themes in this study are suggestive of previously identified characteristics of prescription and over-the-counter medicine addiction, including the initial legitimate reason for starting codeine, which has also been identified among over-the-counter medicine misuse [6]. Posts also described the change in use and exploitation of what were perceived as positive effects, such as a “fuzzy effect” and euphoria; these data share similarities with some of the trip narratives described in more recreational drug use online posts [13]. Similarly, experiences of negative side effects also reflected those previously reported [7,32]. Strategies to address the problem also reflected what previous research has identified. Both individual and informal approaches, such as gradually reducing use or stopping use immediately, appeared most commonly as what had been tried or what had been suggested by others. Of note was that many suggestions were made about seeking formal addiction treatment support but with concerns from original posters about confidentiality [6].

Although this study did not seek to assess the quality of advice provided, there appeared to be variation in the quantity and type of treatment advice provided. However, there was also evidence of what have been termed *counterbalancing comments*, which have been argued to offset poor-quality advice; these have been identified in other online sites for health conditions [18]. However, given the relative absence of updates and conclusions to the original poster’s narratives, it is not possible to comment on the degree to which the original poster acted on the advice given. This finding, along with original posters initially seeking to use such public online sites as well as explicit concerns about not wanting to be identified via formal services, suggests that more still needs to be done to best support individuals affected by codeine, particularly in terms of treatment options. Concerns about existing treatment and support have emerged in previous research in relation to codeine and other medicines [6,33]. While this study does not offer specific recommendations for treatment change, it suggests that these online sites may represent important opportunities to provide support and their use should not be underestimated.

This research has additional implications for practice and further research. A key implication relates to the need to raise awareness among patients and the public regarding the harms related to codeine. A second implication and related recommendation is that online resources could be used more; there may be potential for online forums to be monitored and for more active support to be offered based on the content posted. Research involving nonsuicidal self-injury and analysis of posts about this topic on sites such as Instagram revealed that searches could detect problematic posts and that current warnings provided could be further enhanced [34]. There are opportunities for online forums to consider adding such warnings based on keywords relating to codeine addiction and other implicated medicines. Further research would be needed to consider the benefits and acceptability of such schemes. With regard to digital recovery, this research also has implications for how individuals affected by medicine misuse may gain benefits from social media sites by obtaining treatment or recovery information, as previous research has suggested [22]. This would also require further research and evaluation, particularly to address this study’s findings of *absent endings*.

### Conclusions

Codeine is an important analgesic available both by prescription and over-the-counter in the United Kingdom and around the world. This study offers further evidence that the use of codeine can become problematic; caution is needed in relation to prescribing and supplying codeine as well as in being vigilant in changes in use among patients. This study provides a unique additional insight into how a popular public internet forum can be used by individuals to explore problematic codeine use. In particular, the interactive nature of the forum offers a range of opportunities for others to provide advice and support. However, while this was often very positive, some negative comments emerged and treatment advice also varied. Self-reported codeine addiction was associated with considerable negativity in terms of the harms and side effects reported, the impact it had on individuals, and the associated blame and stigma. Increasing understanding of this issue is still needed for both health professionals and the public, but there may be unique opportunities in the online forums themselves to offer additional support and information. Uncertainty about treatment options suggests that more guidance and support is needed for those affected to help them overcome their problems.

## Abbreviations

GP: general practitioner
NHS: National Health Service
